# Compliance with Two Vaccination Regimens of Freeze-Dried Human Rabies Vaccine (Vero Cell) and Influencing Factors in Hubei Province, China

**DOI:** 10.3390/vaccines14100856

**Published:** 2026-09-28

**Authors:** Dan Li, Haiyan Liang, Debao Li, Shuyu Guo, Xuefa Zheng, Shaohua Liu, Jingjing Kong, Siquan Wang, Zhao Wang, Zhao Jiang, Xianfeng Tang, Jinjun Luo, Ning Li, Luyu Ren, Yan Liu, Zhiang Wu, Lei Wang, Yeqing Tong

**Affiliations:** 1 Hubei Provincial Center for Disease Control and Prevention, Wuhan 430079, China; 2 Shandong Key Laboratory of Human Vaccine Research and Development (in Preparation), Dongying 257091, China; 3 Shandong Yidu Biotechnology Co., Ltd., Dongying 257091, China; 4 Shandong Center for Food and Drug Evaluation and Inspection, Jinan 250014, China; 5 Beijing Yidu Zhengkang Health Technology Co., Ltd., Beijing 100073, China

**Keywords:** rabies vaccine, vaccination compliance, four-dose (Zagreb) rabies vaccination regimen, five-dose (Essen) rabies vaccination regimen

## Abstract

**Objective:** To systematically analyze the practical application and differences in vaccination compliance of two rabies post-exposure prophylaxis regimens based on real-world data, and to explore the associated influencing factors of vaccination compliance, aiming to provide evidence for optimizing vaccination strategies. **Methods:** Vaccination records of exposed individuals who received the freeze-dried human rabies vaccine (Vero cell) documented in the Hubei Provincial Immunization Information Management System from 1 January to 31 December 2024 were retrospectively collected to evaluate compliance with the five-dose (Essen) and four-dose (Zagreb) vaccination schedules. Good compliance was defined as completing the vaccination according to the schedule within the prescribed time frame. The evaluation included the vaccination compliance rate and the full-course vaccination rate. Factors associated with compliance were explored using univariate and multivariable analyses. **Results:** A total of 51,066 subjects were enrolled. The four-dose (Zagreb) schedule was associated with significantly higher vaccination compliance (77.1% vs. 72.0%, *χ*^2^ = 94.913, *p* < 0.05) and full-course completion rates (85.2% vs. 82.4%, *χ*^2^ = 38.251, *p* < 0.05), whereas the five-dose (Essen) regimen was associated with a higher rate of timely initial vaccination (81.7% vs. 78.1%, *χ*^2^ = 61.602, *p* < 0.05). In the multivariable analysis, the four-dose (Zagreb) regimen was also associated with increased full-course completion (aOR = 1.49, 95% CI = 1.39–1.60) and better compliance (aOR = 1.52, 95% CI = 1.43–1.61), whereas in a pre-specified, descriptive exploratory model, it was associated with delayed first doses (exploratory aOR = 0.84, 95% CI = 0.79–0.89); this adjusted association should not be interpreted as a causal effect of regimen on timely initiation. Younger age, lower-limb wounds, high-security occupations, farming, passive immunization and non-winter exposure were associated with better full-course vaccination; adults aged ≥18 years, males, Han/Miao ethnicity, Grade III exposure and cat bites were associated with lower compliance (*p* < 0.05). Severe exposure, farmer/student/resident identity, passive immunization and spring exposure were associated with more timely initiation, whereas male sex, neck-and-above exposure and flexible work were associated with less timely initiation (*p* < 0.05). **Conclusions:** In this retrospective, non-randomized study, receipt of the four-dose (Zagreb) regimen was associated with modestly higher full-course completion (absolute difference: 2.8 percentage points) and better compliance (5.1 percentage points) than the five-dose (Essen) regimen. Conversely, it was associated with a lower rate of timely first-dose vaccination. As an observational study, these findings do not establish causal relationships. Therefore, immediate wound care, prompt vaccination, and appropriate administration of rabies immunoglobulin remain the principal clinical priorities.

## 1. Introduction

Rabies is a zoonotic disease caused by infection with the rabies virus. Once clinical symptoms appear, rabies is virtually 100% fatal [1], and no specific therapeutic agent is currently available. Post-exposure prophylaxis (PEP) is the most effective means of preventing rabies after exposure [1]. The World Health Organization (WHO) considers that timely, scientific, and thorough PEP can prevent the occurrence of rabies [1]. Therefore, both the WHO and the Chinese Center for Disease Control and Prevention recommend rabies vaccination as an important disease control strategy [1,2]. Currently, intramuscular injection is the primary method of rabies vaccination worldwide, although the WHO recommends transitioning from intramuscular to intradermal administration where adequate training and vaccine supply are available [3]. This dose-sparing alternative is not yet approved for routine use in China, where intramuscular regimens remain the only licensed option for post-exposure prophylaxis [2]. In China, licensed intramuscular PEP regimens include the traditional five-dose Essen schedule (one dose on days 0, 3, 7, 14, and 28) and the four-dose Zagreb (2–1–1) schedule (two doses on day 0 and one dose each on days 7 and 21). The WHO currently recommends dose- and time-sparing PEP schedules, while previously recommended regimens remain valid. Although the two regimens have shown comparable immunogenicity and clinical trials have shown the Zagreb regimen to be non-inferior to the Essen regimen, differences in compliance in real-world settings still require thorough comparison [4,5,6,7].

Globally, improving patient compliance with rabies vaccination is a key strategy for rabies management [8]. Studies have shown that among patients receiving standardized rabies post-exposure prophylaxis (PEP), a substantial proportion exhibit poor compliance. Some patients fail to complete the vaccination series due to the financial burden of treatment, while an even larger number demonstrate insufficient awareness of the importance of rabies vaccination, often citing forgetfulness or lack of time for clinic visits [9,10,11,12,13]. However, research on compliance remains limited, particularly regarding large-sample investigations, which hinders systematic exploration of potential factors influencing compliance rates. This retrospective, non-randomized observational study aims to compare compliance and full-course completion rates between four-dose and five-dose vaccination schedules, to explore potential influencing factors, and to provide evidence to inform clinical practice and public health strategies.

## 2. Materials and Methods

### 2.1. Research Subjects

This retrospective study included data on exposed subjects who received the freeze-dried human rabies vaccine (Vero cell) produced by Shandong Yidu Biotechnology Co., Ltd., Dongying, China with records collected from the Hubei Provincial Immunization Information Management System from 1 January 2024 to 31 December 2024. Individuals were included if they had received only the rabies vaccine manufactured by Shandong Yidu Biotechnology Co., Ltd.; were first-time vaccinees (i.e., in a state of first exposure) at the time of the first dose, with the first-dose administered on or after 1 January 2024; and had either completed all doses by the data collection date (31 December 2024) or been recorded as non-compliant according to the study definition. Subjects were divided into the 4-dose (Zagreb) group and the 5-dose (Essen) group according to the actual vaccination regimen they received. The freeze-dried human rabies vaccine (Vero cell) used in this study is approved for both the Essen and Zagreb regimens in China.

### 2.2. Methods

This retrospective study collected and analyzed vaccination data recorded in the Hubei Provincial Immunization Information Management System to compare compliance between the 4-dose and 5-dose rabies vaccination regimens, as well as the associated influencing factors. The study strictly adhered to ethical principles, and all data were anonymized to protect personal privacy. We assessed key indicators, including vaccination timing, dose number, and completion status, and incorporated multiple potential influencing factors, such as age, sex, ethnicity, exposure site, exposure grade, occupation, social identity, offending animal, use of antiserum or immunoglobulin, and season of exposure, to provide evidence for optimizing vaccination strategies.

In this study, different analysis sets were used for the main and sensitivity analyses (terminology adapted from clinical trial methodology and applied here to an observational dataset):

**Full Analysis Set (FAS):** All subjects who met the inclusion criteria.

**Per-Protocol Set 1 (PPS1):** Subjects who met the inclusion criteria and had no missing vaccination time points. This analysis set was used for the sensitivity analysis of vaccination compliance.

**Per-Protocol Set 2 (PPS2):** Subjects who met the inclusion criteria and had no missing vaccination dates. This analysis set was pre-specified as a sensitivity analysis to assess whether the primary results for full-course vaccination would be affected by incomplete date documentation.

**Per-Protocol Set 3 (PPS3):** Subjects who met the inclusion criteria and had no missing data on time of exposure or time of first dose. This analysis set was used for the sensitivity analysis of the timely first-dose vaccination rate.

The STROBE checklist for this study is available as Supplementary Table S2.

### 2.3. Definitions

#### 2.3.1. Compliance

Definition of good compliance with the 4-dose rabies vaccination regimen: Vaccination was administered according to the 4-dose regimen on days 0, 7, and 21. For the second dose on day 0, discontinuation of vaccination was considered non-compliance for that dose. For the third dose (day 7), a delay of more than 48 h beyond the scheduled date or failure to receive that dose was considered non-compliance. For the fourth dose (day 21), a delay of more than 72 h or discontinuation was considered non-compliance for that dose. Good compliance was defined as the absence of any non-compliance for all four doses in a given exposed subject.

Definition of good compliance with the 5-dose rabies vaccination regimen: Vaccination was administered according to the 5-dose regimen on days 0, 3, 7, 14, and 28. For the second dose (day 3), a delay of more than 24 h beyond the scheduled date or failure to receive that dose was considered non-compliance for that dose. For the third and fourth doses (days 7 and 14), a delay of more than 48 h or discontinuation was considered non-compliance for that dose. For the fifth dose (day 28), a delay of more than 72 h or discontinuation was considered non-compliance for that dose. Good compliance was defined as the absence of non-compliance for all five doses in a given exposed subject.

The permissible delay thresholds for both regimens (>24 h for Essen day 3, >48 h for Essen days 7 and 14, >72 h for Essen day 28; failure to receive the second dose on Zagreb day 0, >48 h for Zagreb day 7, >72 h for Zagreb day 21) are operational definitions adopted for this study. These thresholds are consistent with the out-of-window criteria used in previous real-world compliance studies of rabies vaccination in China [11,12] and are compatible with the principle stipulated in the Chinese Specification for Rabies Post-Exposure Prophylaxis (2023 Edition) that delayed doses should be postponed accordingly without restarting the immunization course [2].

Vaccination compliance rate was defined as the proportion of exposed subjects with good compliance among all enrolled subjects. The analysis was conducted using the FAS and the per-protocol sets PPS1, PPS2, and PPS3.

#### 2.3.2. Full-Course Vaccination

For the 4-dose regimen group, full-course vaccination was defined as an exposed subject receiving two doses on day 0 and one dose each on days 7 and 21 as specified, completing a total of four doses. For the 5-dose regimen group, full-course vaccination was defined as an exposed subject receiving one dose each on days 0, 3, 7, 14, and 28 as specified, completing a total of five doses. Subjects who completed all required doses for their respective regimens were considered to have received full-course vaccination. 

The full-course vaccination rate was defined as the proportion of exposed subjects who completed full-course vaccination in the full analysis set (FAS). The analysis was conducted using the FAS and PPS2.

#### 2.3.3. Exposure Grade

Rabies exposure was classified into three grades according to the Chinese Specification for Rabies Post-Exposure Prophylaxis (2023 Edition):

Grade I: Touching or feeding animals or licking of intact skin; no medical intervention is required.

Grade II: Nibbling of exposed skin or minor scratches or abrasions without bleeding; wound care and rabies vaccination are required.

Grade III: Single or multiple transdermal bites or scratches (accompanied by bleeding), licking of broken skin, contamination of open wounds or mucous membranes with saliva or tissue, or direct contact with bats; wound care, rabies passive immunoprophylaxis (rabies antiserum or human rabies immunoglobulin) and rabies vaccination are required.

#### 2.3.4. Timely First-Dose Vaccination

Timely first-dose vaccination was defined as administration of the first rabies vaccine dose within 24 h after the exposure event. This 24 h threshold represents an operational definition adopted for this study, aligned with the principle of “the sooner, the better” stipulated in the Chinese Specification for Rabies Post-Exposure Prophylaxis (2023 Edition) and consistent with the timely vaccination window used in previous real-world rabies vaccine compliance studies in China [11,12]. For this analysis, “day 0” refers to the date of first vaccination, which typically coincided with or immediately followed the exposure date. Doses administered earlier than the scheduled date were not classified as non-compliant for the purpose of this outcome.

#### 2.3.5. Regimen Selection and Temporal Considerations

Both the 4-dose Zagreb and 5-dose Essen regimens were available at all participating immunization clinics in Hubei Province throughout 2024. At the first visit, attending clinicians informed exposed individuals about the available regimens, and the final choice was made by the vaccine recipient. Regimen selection may have been influenced by multiple factors, including clinicians’ recommendations, exposure severity, and exposed individuals’ preferences regarding cost and convenience. Regimen selection and the first-dose administration occurred on the same day; for the Zagreb regimen, two doses were administered on day 0 (left and right deltoid muscles). Baseline characteristics of the two groups are compared in Table S1. The timely first-dose vaccination analysis (Section 3.4) is reported as a descriptive, exploratory comparison between regimen groups because regimen selection preceded or coincided with first-dose administration; consequently, no causal interpretation is made, and the adjusted association with the regimen should not be interpreted as an effect or determinant of timely initiation.

### 2.4. Statistical Analysis

Statistical analyses were performed using SAS version 9.4 (SAS Institute Inc., Cary, NC, USA). Categorical data were compared using the chi-square test or Fisher’s exact test as appropriate. Because regimen selection occurred before or at the time of first-dose administration, the timely first-dose model was considered descriptive and exploratory, and its results should not be interpreted as causal effects (see Section 2.3.5 for rationale). The following covariates were included simultaneously in all models: age, sex, ethnicity, exposure site, exposure grade, occupation, social identity, offending animal, use of antiserum or immunoglobulin, and season. Univariable logistic regression was performed for descriptive purposes. Results are presented as odds ratios (ORs) with their 95% confidence intervals (CIs), which were calculated using the Wald normal approximation method. A two-sided *p*-value ≤ 0.05 was considered statistically significant. For the full-course vaccination analysis, the denominator was restricted to subjects whose scheduled final dose fell on or before 31 December 2024.

The overall likelihood ratio test is reported as a global test of model fit and is not intended to serve as a substitute for assessment of model calibration, discrimination, or influential observations.

Univariable logistic regression results for each outcome are provided in the Supplementary Materials (Tables S3–S5).

Formal collinearity assessment was performed using variance inflation factors (VIFs) for all multivariable models; the generalized variance inflation factor (GVIF) with exponent 1/(2 × df) was calculated for each predictor, and values close to 1.0 were considered indicative of negligible multicollinearity.

## 3. Results

### 3.1. Subject Distribution

A total of 51,066 subjects who met the inclusion criteria were enrolled in this study, all of whom were included in the full analysis set (FAS), with 8589 and 42,477 cases in the four-dose and five-dose regimen groups, respectively. Three per-protocol analysis sets (PPS1, PPS2, and PPS3) were derived. In the four-dose regimen group, 8192, 8589, and 7190 cases were enrolled in PPS1, PPS2, and PPS3, respectively; in the five-dose regimen group, 41,056, 42,477, and 37,449 cases were enrolled in PPS1, PPS2, and PPS3, respectively (Figure 1 and Figure 2). In the FAS, the four-dose and five-dose regimen groups included 4562 and 22,673 males (53.1% and 53.4%) and 4027 and 19,804 females (46.9% and 46.6%), respectively. The age ranged from 0 to 100 years, with a median age of 32.8 years for males and 36.5 years for females.

Baseline characteristics of the Zagreb and Essen groups, including standardized mean differences (|SMD|), are presented in Table S1.

### 3.2. Vaccination Compliance and Influencing Factors

Under the three analysis sets, the four-dose regimen group consistently showed higher compliance rates than the five-dose group (FAS: 77.1% vs. 72.0%, *χ*^2^ = 94.913, *p* < 0.05; PPS1: 76.8% vs. 71.8%, *χ*^2^ = 84.315, *p* < 0.05; PPS2: 84.6% vs. 82.6%, *χ*^2^ = 17.967, *p* < 0.05) (Table 1).

In the multivariable analysis, receipt of the four-dose Zagreb regimen was associated with higher vaccination compliance (aOR = 1.52, 95% CI = 1.43–1.61) compared with the five-dose Essen regimen. Older age, male sex, Han ethnicity, Grade III exposure, cat-inflicted injuries, and refusal of passive immunization were associated with lower compliance, whereas lower-limb exposure, receipt of passive immunization, and high-security/structured occupations were associated with higher compliance. Detailed adjusted estimates for all covariates are provided in Table 2.

### 3.3. Full-Course Vaccination Rate and Influencing Factors

In the primary analysis (FAS), the four-dose group achieved higher full-course vaccination rates than the five-dose group (85.2% vs. 82.4%; *χ*^2^ = 38.251, *p* < 0.05) (Table 3). A pre-specified sensitivity analysis (PPS2), which excluded subjects with missing vaccination dates, was conducted to evaluate the potential impact of such missingness. Because all subjects had complete date records, no participants were excluded relative to the primary analysis; consequently, the pre-specified sensitivity analysis for missing vaccination dates was non-informative, yielding identical results (Table 3).

Multivariable analysis showed that the four-dose regimen was associated with a higher likelihood of full-course vaccination (aOR = 1.49, 95% CI = 1.39–1.60). Consistent with the compliance analysis, older age, male sex, Han ethnicity, cat-inflicted injuries, and refusal of passive immunization were associated with lower full-course rates, whereas lower-limb exposure, receipt of passive immunization, farmer identity, and high-security/structured occupations were associated with higher full-course rates. Full model results are shown in Table 4.

### 3.4. Timely First-Dose Vaccination Rate and Influencing Factors

As regimen selection occurred before or contemporaneously with first-dose administration, the timely first-dose analysis is reported as a descriptive comparison. The four-dose group had lower timely first-dose rates than the five-dose group in both the primary analysis (FAS: 78.1% vs. 81.7%, *χ*^2^ = 61.602, *p* < 0.05) and sensitivity analysis (PPS3: 90.3% vs. 91.4%, *χ*^2^ = 8.907, *p* < 0.05) (Table 5).

In an exploratory multivariable model, for which regimen selection preceded or coincided with first-dose administration and therefore the adjusted association must not be interpreted as a regimen effect on timely initiation, the four-dose regimen remained associated with lower odds of timely first-dose vaccination (exploratory aOR = 0.84, 95% CI = 0.79–0.89). Male sex, neck-and-above exposure, and low-security/flexible occupations were associated with lower timely vaccination, whereas Grade II/III exposures, farmer/student/resident identities, and receipt of passive immunization were associated with higher timely rates. These adjusted estimates are reported for descriptive, hypothesis-generating purposes only and should not be interpreted as causal effects of regimen on timely initiation. Detailed results are provided in Table 6.

## 4. Discussion

Based on a retrospective analysis of real-world data from 51,066 post-exposure subjects in Hubei Province, this study found that receipt of the four-dose Zagreb regimen was associated with higher vaccination compliance and full-course completion compared with the five-dose Essen regimen, whereas the five-dose regimen was associated with a higher rate of timely first-dose vaccination. The absolute differences were modest: approximately 5.1 percentage points for compliance and 2.8 percentage points for full-course completion favoring the Zagreb regimen and approximately 3.6 percentage points for timely first-dose vaccination favoring the Essen regimen. Factors associated with compliance in this study included age, sex, exposure site, exposure grade, ethnicity, occupation and social identity, offending animal, use of antiserum or immunoglobulin, and season of exposure. Timely and complete vaccination is essential for preventing rabies-related deaths. The conventional five-dose regimen faces adherence challenges in real-world settings due to its multiple injections and prolonged schedule [14,15,16].

Good compliance was observed in 77.1% and 72.0% of subjects in the four-dose and five-dose groups, respectively, indicating that the four-dose regimen was associated with better compliance, which is consistent with previous studies [17,18]. Some studies have found a linear relationship between vaccination schedule duration and completion rate: the longer the vaccination schedule, the lower the proportion of individuals completing the vaccination [16,19]. Indeed, a previous study using the same vaccine product reported that the Zagreb regimen had a significantly lower rate of out-of-window administration compared with the five-dose regimen (8.41% vs. 16.38%, *p* < 0.05) [12].

Multivariable analysis revealed that the four-dose regimen group had significantly higher compliance. Factors such as age ≤ 18 years, lower-limb exposure, use of antiserum and immunoglobulin, and exposure during spring, summer, or autumn were associated with improved compliance. Of particular note is the fact that compliance was lower among individuals with Grade III exposure, whereas individuals with lower-limb exposure showed relatively higher compliance. Analysis of exposure characteristics revealed that Grade III exposure predominantly involved the head, face, or upper extremities. According to the standard post-exposure prophylaxis (PEP) protocol, these severe injuries mandate combined administration of rabies vaccine and either anti-rabies serum or rabies immunoglobulin, resulting in substantially higher total treatment costs. This financial burden may lead patients with limited economic resources to delay or discontinue follow-up doses after the initial vaccination. In clinical practice, lower-limb exposures are more likely to be classified as Grade II rather than Grade III, typically requiring vaccination alone without the additional cost of rabies immunoglobulin. This lower financial burden may contribute to the better adherence observed among patients with lower-limb exposures; however, this mechanistic explanation remains hypothetical and should be interpreted as such rather than a demonstrated finding. Consequently, such patients may exhibit better adherence to the complete vaccination schedule. The four-dose regimen effectively reduces the number of visits to healthcare facilities and the number of vaccine doses required; while this likely lowers the risk of dropout, the direct contribution of these cost- and convenience-related factors to the observed adherence differences is hypothesized rather than empirically demonstrated in the present dataset. This hypothesis is supported by health-economic evidence across multiple lines. A seminal cost-effectiveness model demonstrated that regimens requiring fewer clinic visits incur lower indirect costs and that prioritizing reduced-visit regimens is essential for optimizing the delivery of post-exposure prophylaxis, under the explicit assumption that higher out-of-pocket costs reduce patient compliance [20]. Corroborating this, a 2024 systematic review of One-Health interventions for rabies elimination identified the efficient use of PEP combined with a shift toward abridged vaccination schedules as a core component of the most cost-effective elimination programs [21]. Further reinforcing the economic imperative, a 2024 provincial-level economic analysis in Vietnam revealed that post-exposure treatment accounts for 84.5% of total rabies control expenditure, underscoring the substantial financial burden of PEP and the necessity of optimizing its delivery [22]. In the present study, however, direct cost data (individual income, insurance status, transportation costs, and actual out-of-pocket treatment costs) were not measured; therefore, the potential economic advantage of reduced-visit regimens such as the Zagreb schedule remains a hypothesis that warrants prospective health-economic evaluation.

In the United States, where healthcare resources are more accessible, the primary reasons for incomplete rabies vaccination include patient forgetfulness, lack of time for clinic visits, and high treatment burden [10]. In contrast, poor compliance among patients in developing countries such as those in Southeast Asia, India, and Africa is more likely attributable to insufficient insurance coverage, low socioeconomic status, and poor risk perception [23,24,25]. A study conducted in Shenzhen, China, found that participants aged 31–40 years exhibited even poorer compliance [25], which is consistent with our findings. These observations indicate that compliance with rabies vaccination is influenced by multiple factors; however, the underlying mechanisms warrant further investigation.

In contrast to the above advantages, the timely first-dose vaccination rate within 24 h post-exposure was 81.7% in the five-dose group and 78.1% in the four-dose group, with the five-dose group exhibiting a higher rate. Several hypotheses may explain this finding; however, these explanations are speculative and were not directly tested in the present dataset. First, the five-dose regimen, as a classic protocol, may be more often selected for high-risk exposures where patients have a strong sense of urgency, whereas the four-dose regimen, as a simplified protocol, may be more frequently applied to lower-risk exposures. Second, as a hypothesis, individuals who choose the five-dose regimen may have stronger health awareness, which could enable them to receive the first dose promptly and adhere to the full schedule. This explanation could not be tested directly because individual health literacy, income, insurance status, transportation costs, and actual treatment costs were not measured in this study. Third, individuals who present promptly for vaccination may be more likely to receive the five-dose regimen. The four-dose regimen, with its two doses administered on day 0, has been shown to induce earlier seroconversion and higher antibody titers at day 7 compared with the five-dose regimen [26]. Previous studies have also demonstrated that the Zagreb regimen maintains durable antibody concentrations in the longer term, being non-inferior to the Essen regimen in Vero cell rabies vaccines [27], and that booster vaccination after the Zagreb regimen induces higher antibody titers than after the Essen regimen [28]. This enhanced immunogenicity may offer immunological advantages for individuals with delayed post-exposure prophylaxis initiation. This hypothesis could not be directly tested in the current dataset and requires prospective validation. Notably, the 815 Grade I exposure participants included in this study likely reflect self-requested prophylaxis in routine clinical practice, where attending clinicians typically accommodate patients’ precautionary demands despite guideline recommendations against vaccination for this exposure level.

### Strengths and Limitations

This study has several strengths, including its large sample size drawn from a mandatory electronic immunization registry with complete dose-date documentation and the application of both primary and sensitivity analyses. However, important limitations must be acknowledged. First, subjects were restricted to Hubei Province and received a single vaccine brand, restricting the generalizability of findings. Second, as a retrospective observational study, regimen assignment was not randomized. Patients, clinicians, or facilities may have selected the regimen based on factors that also influence compliance—including health literacy, risk perception, economic circumstances, and transportation access—that were not directly measured in the database. Consequently, residual confounding by indication and self-selection likely persists despite multivariable adjustment, and the adjusted estimates should not be interpreted as causal regimen effects. Third, the database lacked facility-level identifiers, precluding facility-level clustering analysis. Fourth, administrative region could not be included as a covariate due to inconsistent coding and a high proportion of unclassifiable records, potentially leaving residual geographic confounding unaddressed. Fifth, only short-term vaccination compliance was analyzed, without long-term antibody follow-up to verify clinical protective effects. Future research should employ a multicenter prospective cohort study design to more accurately evaluate the relationship between vaccination regimens and adherence and to incorporate long-term immunogenicity assessments, as has been demonstrated in recent prospective studies on antibody persistence following different dosing schedules [29]. Finally, the timely first-dose analysis is descriptive rather than causal, as regimen selection precedes or coincides with first-dose administration.

Several limitations should be acknowledged. First, we did not perform propensity-score weighting or matching, and standardized mean differences (|SMD|) indicated residual baseline imbalance between the Zagreb and Essen groups for several variables (e.g., age, passive immunization status; see Table S1), despite adjustment in the multivariable models. Second, adjusted odds ratios (aORs) may overestimate true prevalence ratios because the outcomes are common (vaccination compliance and full-course completion rates of ~85–90%), and a modified Poisson model with robust variance was not fitted; accordingly, interpretation is anchored primarily on the modest absolute differences (5.1 and 2.8 percentage points for the main compliance outcomes) rather than on the magnitude of the odds ratios. Third, formal collinearity assessment using variance inflation factors (VIFs) was performed for all multivariable models, and all GVIF^(1/(2 × Df)) values were close to 1.0 (range: 1.01–1.22), indicating no significant multicollinearity among the predictors (see Supplementary Material, Table S6). Fourth, all 10 covariates were pre-specified by clinical relevance, but interaction tests for effect modification and adjustments for multiple comparisons were not conducted; thus, the results should be interpreted with caution as average associations, given the potential for type I error. Furthermore, because the registry data could not distinguish between loss to follow-up, non-compliance, and discontinuation of vaccination for other reasons (e.g., adverse events or patient decision), some cases of ‘non-compliance’ may have been misclassified.

## 5. Conclusions

In this retrospective, single-province cohort, receipt of the Zagreb regimen was associated with modestly higher full-course vaccination completion (absolute difference: 2.8 percentage points) and better compliance (5.1 percentage points) than the Essen regimen. Conversely, the Zagreb regimen was associated with a lower timely first-dose rate. As an observational study, these findings do not establish causal relationships between regimen and compliance outcomes. Neither vaccination schedule can compensate for delayed post-exposure prophylaxis initiation. Immediate wound care, prompt vaccination, and appropriate administration of rabies immunoglobulin remain the principal clinical priorities.

## Figures and Tables

**Figure 1:**
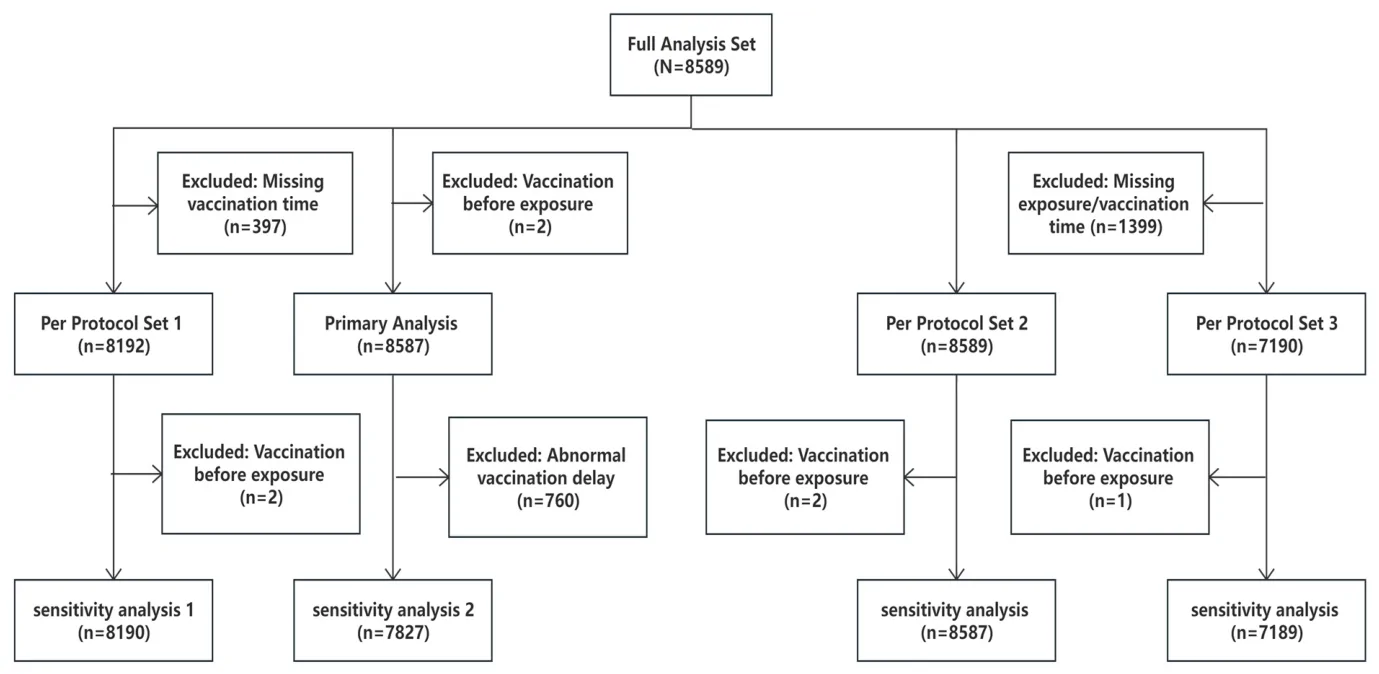
Subject distribution in the 4-dose (Zagreb) rabies vaccination regimen group.

**Figure 2:**
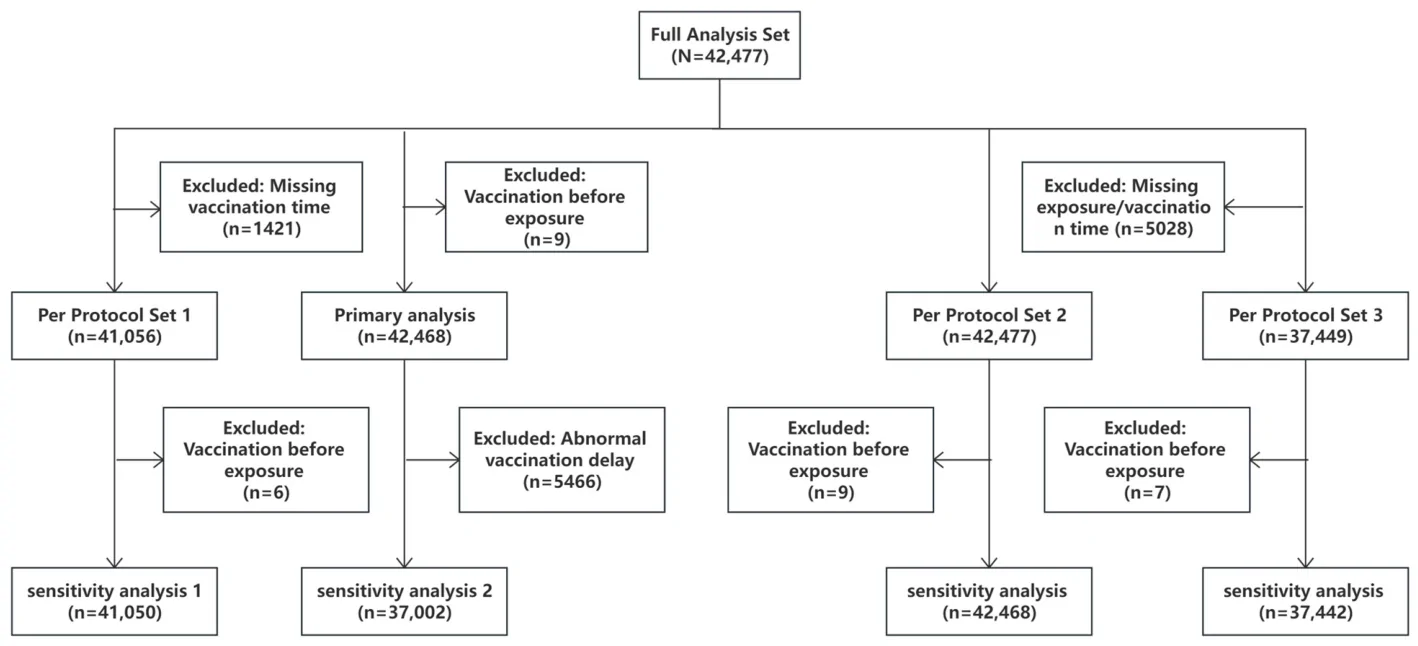
Subject distribution in the 5-dose (Essen) rabies vaccination regimen group.

**Table 1:** The vaccination compliance rates in the 4-dose (Zagreb) and 5-dose (Essen) rabies vaccination regimen groups in Hubei Province, China.

	Primary Analysis Number (%)		Sensitivity Analysis 1 Number (%)		Sensitivity Analysis 2 Number (%)	
	4-Dose Regimen	5-Dose Regimen	4-Dose Regimen	5-Dose Regimen	4-Dose Regimen	5-Dose Regimen
Results	8587	42,468	8190	41,050	7827	37,002
n (missing)	8587 (0)	42,468 (0)	8190 (0)	41,050 (0)	7827 (0)	37,002 (0)
Poor compliance	1968 (22.9)	11,911 (28)	1903 (23.2)	11,572 (28.2)	1208 (15.4)	6445 (17.4)
Good compliance	6719 (77.1)	30,557 (72.0)	6287 (76.8)	29,478 (71.8)	6619 (84.6)	30,557 (82.6)
Difference (95% CI)	5.1 (4.1, 6.1)		5.0 (3.9, 6.0)		2.0 (1.1, 2.9)	
Statistic	94.913		84.315		17.967	
*p*-value	<0.05		<0.05		<0.05	

**Table 2:** Multivariable analyses of vaccination compliance rates in the 4-dose (Zagreb) and 5-dose (Essen) rabies vaccination regimen groups in Hubei Province, China.

Variables	Multivariable N	Adjusted OR (aOR)	Wald 95% CI	*p*-Value
**Treatment regimen**				
5-dose	40,605	Reference	–	–
4-dose	8031	1.52	(1.43, 1.61)	<0.05
**Age (years** **)**				
≤18	12,881	Reference	–	–
[18, 40]	15,092	0.38	(0.35, 0.40)	<0.05
[40, 60]	13,004	0.75	(0.69, 0.81)	<0.05
>60	7659	0.96	(0.87, 1.05)	0.34
**Sex**				
Female	25,819	Reference	–	–
Male	22,817	0.79	(0.76, 0.83)	<0.05
**Ethnicity**				
Other	25,104	Reference	–	–
Han	22,951	0.81	(0.77, 0.84)	<0.05
Miao	100	0.60	(0.39, 0.92)	<0.05
Tujia	481	0.82	(0.66, 1.02)	0.07
**Exposure site**				
Upper limbs and trunk	30,437	Reference	–	–
Lower limbs	16,544	1.14	(1.09, 1.20)	<0.05
Neck and above	1655	0.94	(0.84, 1.05)	0.27
**Exposure grade**				
Grade I	738	Reference	–	–
Grade II	18,244	0.98	(0.81, 1.17)	0.79
Grade III	29,654	0.82	(0.69, 0.99)	<0.05
**Occupational category**				
Other	17,076	Reference	–	–
Medium-security/Market-oriented	7019	1.03	(0.97, 1.10)	0.31
Economically inactive	16,309	0.98	(0.93, 1.04)	0.59
Low-security/Flexible	5616	1.13	(1.04, 1.22)	<0.05
High-security/Structured	2616	1.09	(0.99, 1.20)	0.07
**Social identity**				
Other	27,631	Reference	–	–
Farmer	2904	1.11	(0.99, 1.23)	0.07
Student	5773	0.93	(0.86, 1.00)	0.07
Resident	12,328	0.95	(0.89, 1.01)	0.09
**Offending animal**				
Other or unknown animal	2291	Reference	–	–
Dog	24,444	0.99	(0.89, 1.09)	0.79
Cat	21,901	0.84	(0.76, 0.93)	<0.05
**Use of antiserum or immunoglobulin**				
Not used	21,477	Reference	–	–
Used	15,406	1.76	(1.63, 1.91)	<0.05
Refused	11,529	0.84	(0.78, 0.91)	<0.05
**Exposure season**				
Winter	4920	Reference	–	–
Summer	16,825	1.48	(1.35, 1.61)	<0.05
Spring	12,834	1.73	(1.59, 1.88)	<0.05
Autumn	13,654	1.79	(1.64, 1.96)	<0.05

For passive immunization: ‘Not used’ indicates that no passive immunization was recorded as administered (reason not specified in the database); ‘Used’ indicates that rabies immunoglobulin or antiserum was administered; ‘Refused’ indicates that the patient or guardian explicitly declined administration. Model fit: The multivariable logistic regression model was significant overall (likelihood ratio test: *χ*^2^ = 2946.310, *p* < 0.001).

**Table 3:** Differences in full-course vaccination rates in the 4-dose (Zagreb) and 5-dose (Essen) rabies vaccination regimen groups in Hubei Province, China.

	Primary Analysis		Sensitivity Analysis	
	4-Dose Regimen	5-Dose Regimen	4-Dose Regimen	5-Dose Regimen
Results	8587	42,468	8587	42,468
The theoretical/actual administration date of the final dose was no later than 31 December 2024	8552	42,078	8552	42,078
The theoretical/actual date of the last dose was later than 31 December 2024	35	390	35	390
Number of completed full course (%)	7287 (85.2)	34,692 (82.4)	7287 (85.2)	34,692 (82.4)
Number of incomplete vaccination (%)	1265 (14.8)	7386 (17.6)	1265 (14.8)	7386 (17.6)
Difference (95% CI)	2.8 (1.9, 3.6)		2.8 (1.9, 3.6)	
Statistic	38.251		38.251	
*p*-value	<0.05		<0.05	

Denominators included only subjects whose theoretical or actual final dose date was on or before December 31, 2024. Subjects with a scheduled final dose beyond the cutoff were excluded. FAS: full analysis set; PPS2: per-protocol Set 2 (subjects with no missing vaccination dates). PPS2 results are identical to FAS because all subjects in the FAS had complete vaccination date records.

**Table 4:** Multivariable analyses of factors associated with full-course vaccination rate in the 4-dose (Zagreb) and 5-dose (Essen) rabies vaccination regimen groups in Hubei Province, China.

Variables	Multivariable N	Adjusted OR (aOR)	Wald 95% CI	*p*-Value
**Treatment regimen**				
5-dose	40,217	Reference	–	–
4-dose	7996	1.49	(1.39, 1.60)	<0.05
**Age (years** **)**				
≤18	12,798	Reference	–	–
[18, 40]	14,886	0.27	(0.25, 0.29)	<0.05
[40, 60]	12,907	0.53	(0.48, 0.59)	<0.05
>60	7622	0.56	(0.50, 0.63)	<0.05
**Sex**				
Female	25,610	Reference	–	–
Male	22,603	0.74	(0.70, 0.77)	<0.05
**Ethnicity**				
Other	24,898	Reference	–	–
Tujia	477	0.82	(0.62, 1.08)	0.16
Han	22,738	0.76	(0.72, 0.81)	<0.05
Miao	100	0.47	(0.30, 0.76)	0.05
**Exposure site**				
Upper limbs and trunk	30,104	Reference	–	–
Lower limbs	16,479	1.16	(1.09, 1.23)	<0.05
Neck and above	1630	0.88	(0.77, 1.01)	0.08
**Exposure grade**				
Grade I	733	Reference	–	–
Grade II	18,097	0.97	(0.78, 1.21)	0.80
Grade III	29,383	0.77	(0.61, 0.96)	<0.05
**Occupational category**				
Other	16,928	Reference	–	–
Medium-security/Market-oriented	6935	1.07	(0.99, 1.16)	0.09
Low-security/Flexible	5581	1.08	(0.99, 1.18)	0.1
Economically inactive	16,173	0.94	(0.88, 1.01)	0.09
High-security/Structured	2596	1.20	(1.07, 1.34)	<0.05
**Social identity**				
Other	27,483	Reference	–	–
Farmer	2881	1.18	(1.05, 1.34)	<0.05
Student	5711	1.01	(0.92, 1.12)	0.79
Resident	12,138	1.10	(1.02, 1.18)	0.01
**Offending animal**				
Other or unknown animal	2262	Reference	–	–
Dog	24,273	0.92	(0.81, 1.04)	0.19
Cat	21,678	0.80	(0.71, 0.91)	<0.05
**Use of antiserum/immunoglobulin**				
Not used	21,736	Reference	–	–
Used	15,039	1.77	(1.64, 1.92)	<0.05
Refused	11,438	0.82	(0.76, 0.88)	<0.05
**Exposure season**				
Winter	4920	Reference	–	–
Summer	16,821	1.49	(1.36, 1.63)	<0.05
Spring	12,828	1.74	(1.60, 1.89)	<0.05
Autumn	13,644	1.81	(1.65, 1.98)	<0.05

Model fit: The multivariable logistic regression model was significant overall (likelihood ratio test: *χ*^2^ = 2630.026, *p* < 0.001).

**Table 5:** Differences in timely first-dose vaccination rates in the 4-dose (Zagreb) and 5-dose (Essen) rabies vaccination regimen groups in Hubei Province, China.

	Primary Analysis		Sensitivity Analysis	
	4-Dose Regimen	5-Dose Regimen	4-Dose Regimen	5-Dose Regimen
Results	8587	42,468	7189	37,442
n (missing)	8587 (0)	42,468 (0)	7189 (0)	37,442 (0)
Timely vaccination (%)	6703 (78.1)	34,695 (81.7)	6494 (90.3)	34,229 (91.4)
Non-timely vaccination (%)	1884 (21.9)	7773 (18.3)	695 (9.7)	3213 (8.6)
Difference (95% CI)	−3.6 (−4.6, −2.7)		−1.1 (−1.8, −0.3)	
Statistic	61.602		8.907	
*p*-value	<0.001		0.003	

**Table 6:** Multivariable analyses of factors influencing timely first-dose vaccination status in the 4-dose (Zagreb) and 5-dose (Essen) rabies vaccination regimen groups in Hubei Province, China.

Variables	Multivariable N	Adjusted OR (aOR)	Wald 95% CI	*p*-Value
**Treatment regimen**				
5-dose	40,605	Reference	–	–
4-dose	8031	0.84	(0.79, 0.89)	<0.05
**Age (years)**				
≤18	12,881	Reference	–	–
[18, 40]	15,092	0.96	(0.89, 1.04)	0.33
[40, 60]	13,004	1.08	(0.99, 1.18)	0.10
>60	7659	1.11	(1.00, 1.23)	<0.05
**Sex**				
Female	25,819	Reference	–	–
Male	22,817	0.94	(0.90, 0.99)	<0.05
**Ethnicity**				
Other	25,104	Reference	–	–
Tujia	481	0.89	(0.71, 1.12)	0.33
Han	22,951	1.00	(0.95, 1.06)	0.93
Miao	100	2.33	(1.17, 4.64)	0.02
**Exposure site**				
Upper limbs and trunk	30,437	Reference	–	–
Lower limbs	16,544	1.02	(0.97, 1.08)	0.39
Neck and above	1655	0.82	(0.72, 0.92)	<0.05
**Exposure grade**				
Grade I	738	Reference	–	–
Grade II	18,244	1.90	(1.63, 2.22)	<0.05
Grade III	29,654	2.28	(1.94, 2.69)	<0.05
**Occupational category**				
Other	17,076	Reference	–	–
Medium-security/Market-oriented	7019	1.00	(0.92, 1.08)	0.94
Low-security/Flexible	5616	0.82	(0.76, 0.89)	<0.05
Economically inactive	16,309	0.94	(0.88, 1.01)	0.89
High-security/Structured	2616	0.94	(0.84, 1.05)	0.28
**Social identity**				
Other	27,631	Reference	–	–
Farmer	2904	1.31	(1.17, 1.46)	<0.05
Student	5773	1.29	(1.18, 1.41)	<0.05
Resident	12,328	1.33	(1.24, 1.43)	<0.05
**Use of antiserum/immunoglobulin**				
Not used	21,925	Reference	–	–
Used	15,120	1.43	(1.33, 1.55)	<0.05
Refused	11,591	1.28	(1.19, 1.37)	<0.05
**Exposure season**				
Winter	5314	Reference	–	–
Summer	16,822	0.98	(0.90, 1.07)	0.71
Spring	12,829	1.36	(1.25, 1.47)	<0.05
Autumn	13,671	1.02	(0.93, 1.11)	0.67

Model fit: The multivariable logistic regression model was significant overall (likelihood ratio test: *χ*^2^ = 784.632, *p* < 0.001).

## Data Availability

Data analyzed in the present work are not open-access because they contain sensitive individual information that could threaten participants’ privacy and are subject to restrictions of the funding agreement. Relevant data can be provided by the corresponding author upon reasonable request.

## Supplementary Materials

The following supporting information can be downloaded at: https://www.mdpi.com/article/10.3390/vaccines14100856/s1, Table S1: Baseline characteristics of study participants by vaccination regimen (Zagreb vs. Essen); Table S2: STROBE Checklist for observational studies [30]; Table S3: Univariable analysis of factors associated with vaccination compliance; Table S4: Univariable analysis of factors associated with full-course vaccination completion; Table S5: Univariable analysis of factors associated with timely first-dose vaccination; Table S6: Variance inflation factors (VIFs) for predictors in the multivariable logistic regression models.
