# Supplementary material for: Compliance with Two Vaccination Regimens of Freeze-Dried Human Rabies Vaccine (Vero Cell) and Influencing Factors in Hubei Province, China
Source: Vaccines (Basel). 2026 Sep 28;14(10):856. doi: 10.3390/vaccines14100856 (PMC13709589; doi:10.3390/vaccines14100856)
Supplement: Supplementary file 1 [file vaccines-14-00856-s001.zip › vaccines-4474040-supplementary.pdf]

Supplementary Material to:

Compliance with Two Vaccination Regimens of Freeze-dried Human Rabies Vaccine (Vero Cell) and Influencing Factors in Hubei Province, China

Corresponding author: Zhi'ang Wu, wuzhiang@sdydbio.com; Yeqing Tong, t_yeqing@163.com

Contents

Table S1. Baseline characteristics of study participants by vaccination regimen (Zagreb vs. Essen).
Table S2. STROBE Checklist for observational studies.
Table S3. Univariable analysis of factors associated with vaccination compliance.
Table S4. Univariable analysis of factors associated with full-course vaccination completion.
Table S5. Univariable analysis of factors associated with timely first-dose vaccination.
Table S6. Variance inflation factors (VIF) for predictors in the multivariable logistic regression models.

Table S1. Baseline characteristics of study participants by vaccination regimen

Characteristic	Zagreb group (n=8,589)	Essen group (n=42,477)	|SMD|
**Age, years, n(%)**			0.3210
⩽18	1,299 (15.124)	12,425 (29.251)	0.3210
19-40	3,222 (37.513)	12,537 (29.515)	0.1735
41-60	2,825 (32.891)	10,763 (25.338)	0.1713
＞60	1,243 (14.472)	6,736 (15.858)	0.0382
dropout	0 (0.0)	16 (0.038)	
**Sex, n (%)**			0.0201
Female	4,109 (47.840)	19,895 (46.837)	0.0201
male	4,480 (52.160)	22,566 (53.125)	0.0193
dropout	0 (0.0)	16 (0.038)	
**Ethnicity, n (%)**			0.1669
Han	3,483 (40.552)	20,759 (48.871)	0.1669
Tujia	184 (2.142)	313 (0.737)	0.1434
Miao	17 (0.198)	85 (0.200)	0.0005
Other	75 (0.873)	234 (0.551)	0.0416
dropout	4,830 (56.235)	21,086 (49.641)	
**Exposure site, n (%)**			0.0305
Upper limbs & trunk	5,259 (61.229)	26,486 (62.354)	0.0305
Lower limbs	2,934 (34.160)	13,968 (32.884)	0.0232
Head/neck & above	252 (2.934)	1,481 (3.487)	0.0271
dropout	144 (1.677)	542 (1.276)	
**Exposure grade, n (%)**			0.0848
Grade I	108 (1.257)	707 (1.664)	0.0325
Grade II	3,608 (42.007)	16,090 (37.879)	0.0848
Grade III	4,847 (56.433)	25,635 (60.350)	0.0799
dropout	26 (0.303)	45 (0.106)	
**Occupational category, n(%)**			0.1662
Other	3,149 (36.663)	14,602 (34.376)	0.0480
Medium-security/Market-oriented	1,216 (14.158)	6,187 (14.566)	0.0116
Low-security/Flexible	1,278 (14.879)	4,625 (10.888)	0.1250

Characteristic	Zagreb group (n=8,589)	Essen group (n=42,477)	|SMD|
Economically inactive	2,341 (27.256)	14,911 (35.104)	0.1662
High-security/Structured	605 (7.044)	2,136 (5.029)	0.0895
dropout	0 (0.0)	16 (0.038)	
**Social identity, n (%)**			0.1432
Other	5,026 (58.517)	24,961 (58.764)	0.0050
Farmer	578 (6.730)	2,340 (5.509)	0.0526
Student	650 (7.568)	5,141 (12.103)	0.1432
Resident	2,335 (27.186)	10,019 (23.587)	0.0841
dropout	0 (0.0)	16 (0.038)	
**Offending animal, n (%)**			0.0275
Dog	4,447 (51.776)	21,409 (50.401)	0.0275
Cat	3,726 (43.381)	18,930 (44.565)	0.0238
Other/unknown	375 (4.366)	2,028 (4.774)	0.0193
dropout	41 (0.477)	110 (0.259)	
**Passive immunization, n (%)**			0.2038
Net used	4,063 (47.305)	18,281 (43.037)	0.0861
Used	1,901 (22.133)	13,353 (31.436)	0.2038
Refused	2,197 (25.579)	9,517 (22.405)	0.0755
dropout	428 (4.983)	1,326 (3.122)	
**Season, n (%)**			0.1113
Spring	2,283 (26.581)	11,278 (26.551)	0.0007
Summer	2,580 (30.038)	14,473 (34.073)	0.0856
Autumn	2,301 (26.790)	11,376 (26.782)	0.0002
Winter	1,399 (16.288)	5,322 (12.529)	0.1113
dropout	26 (0.303)	28 (0.066)	

Table S2. STROBE Checklist for observational studies

Item #	STROBE Recommendation	Reported on Page # / Section	Notes
**Title and abstract**			
1	Title and abstract: Indicate the study's design with a commonly used term. Abstract should include objective, methods, results, conclusion.	Page 1 (Abstract)	Title specifies 'Comparative Analysis' and regimens; Abstract has Background, Methods, Results, Conclusion.
**Introduction**			
2	Background/rationale: Explain the scientific background and rationale for the investigation.	Page 2 (Introduction, first paragraph)	Rabies burden, Chinese guidelines, WHO position, immunogenicity of Zagreb vs. Essen.
3	Objectives: State specific objectives, including any prespecified hypotheses.	Page 2 (Introduction, second paragraph)	Objective: compare adherence between 4-dose Zagreb and 5-dose Essen regimens.
**Methods**			
4	Study design: Present key elements of study design early in the paper.	Page 3 (Section 2, first paragraph)	Described as retrospective, non-randomized observational study.
5	Setting: Describe the setting, locations, and relevant dates, including periods of recruitment, exposure, follow-up, and data collection.	Page 3 (Section 2.1)	Hubei Provincial CDC immunization information system; data from Jan 1 to Dec 31, 2024.
6	Participants: Give the eligibility criteria and the sources and methods of selection of participants.	Page 3 (Section 2.1); Figures 1 & 2	Inclusion/exclusion criteria detailed; participant flow in Figures 1 & 2.
7	Variables: Clearly define all outcomes, exposures, predictors, potential confounders, and effect modifiers.	Page 4 (Section 2.3)	Compliance, full-course completion, timely first-dose defined; covariates listed.
8	Data sources/measurement: For each variable, describe the data source and method of assessment.	Pages 3–4 (Sections 2.1, 2.3)	Data from Hubei PIIS; variable definitions provided (e.g., day 0, regimen selection).
9	Bias: Describe any potential sources of bias and how they were addressed.	Page 13 (Strengths and Limitations)	Residual confounding, selection bias, lack of facility identifiers acknowledged.
10	Study size: Explain how the study size was decided.	Page 3 (Section 2.1)	Total N=51,066; all eligible records in the database for 2024.
11	Quantitative variables: Explain how quantitative variables were handled in the analyses.	Page 4 (Section 2.3)	Categorical variables listed with levels; continuous variables (e.g., age) categorized.
12	Statistical methods: Describe all statistical methods, including those used to control for confounding.	Page 5 (Section 2.4)	Multivariable logistic regression; pre-specified covariates; complete-case analysis; sensitivity analyses (PPS2).
**Results**			
13	Participants: Report numbers	Page 6 (Section 3.1; Figures 1	Flow diagrams show FAS

	of individuals at each stage of the study.	& 2)	(51,066), PPS1, PPS2, PPS3.
14	Descriptive data: Give characteristics of study participants and information on exposures.	Page 7; Supplementary Table S1	Table 1 shows compliance by subgroup; Table S1 shows baseline characteristics.
15	Outcome data: Report numbers of outcome events or summary measures.	Page 7, Table 1	Compliance and full-course completion rates reported for each regimen.
16	Main results: Give unadjusted and adjusted estimates, and their precision.	Pages 8–10, Tables 2, 4, 6	aORs with 95% CIs reported; covariate-specific P-values provided; omnibus (likelihood ratio test) P-values to be added in the final version.
17	Other analyses: Report any other analyses done (e.g., sensitivity, subgroup).	Pages 8–10 (Sections 3.2–3.4)	PPS2 sensitivity analysis for full-course completion reported.
**Discussion**			
18	Key results: Summarize key results with reference to study objectives.	Pages 6–11 (Section 3)	Zagreb associated with higher compliance and completion; timely first-dose as descriptive comparison.
19	Limitations: Discuss limitations, considering sources of potential bias.	Page 13 (Strengths and Limitations)	Non-randomized design; residual confounding; no PS weighting; no VIF; Table S1 SMD to be included in final version.
20	Interpretation: Give a cautious overall interpretation, considering objectives, limitations, and other evidence.	Pages 11–13 (Discussion)	Results interpreted as associations, not causal; consistent with existing literature.
21	Generalisability: Discuss the generalisability (external validity) of the study results.	Page 13 (Limitations)	Single-province, single-vaccine-brand data limit generalizability.
**Other information**			
22	Funding: Give the source of funding and the role of funders.	page 13(Funding section)	Confirm against the Funding / Conflicts of Interest statement in the manuscript; use N/A if absent.

Reference: von Elm E, Altman DG, Egger M, et al.; STROBE Initiative. Strengthening the Reporting of Observational Studies in Epidemiology (STROBE) statement: guidelines for reporting observational studies. Lancet. 2007;370(9596):1453-1457.

Table S3. Univariable analysis of factors associated with vaccination compliance

Variables	Univariable logistic regression analysis			
	N	Odds Ratio	Wald 95% CI	P-value
**Treatment regimen**				
5-dose	42,468	Reference		
4-dose	8,587	1.31	(1.24, 1.38)	<0.05
**Age**				<0.05
≤18	13,720	Reference		
(18,40]	15,755	0.45	(0.42, 0.47)	<0.05
(40,60]	13,586	0.99	(0.93, 1.05)	0.70
＞60	7,978	1.31	(1.22, 1.40)	<0.05
**Sex**				<0.05
Female	27,043	Reference		
Male	23,996	0.83	(0.80, 0.86)	<0.05
**Ethnicity**				<0.05
Other	26,218	Reference		
Tujia	497	1.03	(0.84, 1.27)	0.74
Han	24,238	0.88	(0.85, 0.92)	<0.05
Miao	102	0.57	(0.38, 0.85)	<0.05
**Exposure site**				<0.05
Upper limbs and trunk	31,740	Reference		
Lower limbs	16,897	1.41	(1.35, 1.47)	<0.05
Neck and above	1,733	0.99	(0.89, 1.10)	0.86
**Exposure grade**				<0.05
Grade I	815	Reference		
Grade II	19,693	0.85	(0.72, 1.01)	0.07
Grade III	30,476	0.74	(0.63, 0.87)	<0.05
**Occupational category**				<0.05
Other	17,748	Reference		
Medium-security / Market-oriented	7,401	1.04	(0.98, 1.11)	0.15
Low-security / Flexible	5,903	1.51	(1.41, 1.62)	<0.05
Economically inactive	17,246	1.07	(1.03, 1.13)	<0.05
High-security / Structured	2,741	1.04	(0.96, 1.14)	0.34
**Social identity**				<0.05
Other	29,981	Reference		
Farmer	2,917	1.56	(1.42, 1.72)	<0.05
Student	5,788	1.11	(1.04, 1.18)	<0.05
Resident	12,353	0.95	(0.90, 0.99)	<0.05
**Offending animal**				<0.05
Other or unknown animal	2,402	Reference		
Dog	25,850	1.05	(0.96, 1.16)	0.28
Cat	22,652	0.77	(0.70, 0.85)	<0.05
**Use of antiserum or immunoglobulin**				<0.05
Not used	22,339	Reference		
Used	15,252	1.09	(1.04, 1.14)	<0.05
Refused	11,711	0.69	(0.66, 0.73)	<0.05
**Exposure season**				<0.05
Winter	6,720	Reference		
Summer	17,049	1.67	(1.58, 1.78)	<0.05
Spring	13,558	1.89	(1.78, 2.01)	<0.05
Autumn	13,674	1.88	(1.76, 2.00)	<0.05

Table S4. Univariable analysis of factors associated with full-course vaccination completion

Variables	Univariable logistic regression analysis			
	N	Odds Ratio	Wald 95% CI	*P*-value
**Treatment regimen**				
5-dose	42,078	Reference		
4-dose	8,552	1.23	(1.15, 1.31)	<0.05
**Age**				<0.05
≤18	13,637	Reference		
(18,40]	15,548	0.34	(0.32, 0.36)	<0.05
(40,60]	13,489	0.76	(0.71, 0.82)	<0.05
＞60	7,941	0.80	(0.74, 0.87)	<0.05
**Sex**				<0.05
Female	26,833	Reference		
Male	23,782	0.78	(0.75, 0.82)	<0.05
**Ethnicity**				<0.05
Other	26,011	Reference		
Tujia	493	1.22	(0.94, 1.57)	0.13
Han	24,024	0.95	(0.91, 1.00)	<0.05
Miao	102	0.53	(0.34, 0.82)	<0.05
**Exposure site**				<0.05
Upper limbs and trunk	31,407	Reference		
Lower limbs	16,832	1.36	(1.29, 1.43)	<0.05
Neck and above	1,708	1.06	(0.93, 1.20)	0.39
**Exposure grade**				<0.05
Grade I	810	Reference		
Grade II	19,546	0.86	(0.70, 1.06)	0.15
Grade III	30,205	0.73	(0.60, 0.90)	<0.05
**Occupational category**				<0.05
Other	17,599	Reference		
Medium-security / Market-oriented	7,317	1.17	(1.09, 1.25)	<0.05
Low-security / Flexible	5,868	1.34	(1.24, 1.46)	<0.05
Economically inactive	17,110	1.20	(1.13, 1.27)	<0.05
High-security / Structured	2,721	1.16	(1.04, 1.29)	<0.05
**Social identity**				<0.05
Other	29,832	Reference		
Farmer	2,894	1.39	(1.24, 1.55)	<0.05
Student	5,726	1.38	(1.27, 1.50)	<0.05
Resident	12,163	1.03	(0.98, 1.09)	0.23
**Offending animal**				<0.05
Other or unknown animal	2,373	Reference		
Dog	25,679	0.95	(0.84, 1.07)	0.36
Cat	22,429	0.73	(0.65, 0.82)	<0.05
**Use of antiserum or immunoglobulin**				<0.05
Not used	22,150	Reference		
Used	15,171	1.30	(1.23, 1.38)	<0.05
Refused	11,558	0.63	(0.59, 0.66)	<0.05
**Exposure season**				<0.05
Winter	6,325	Reference		
Summer	17,048	1.57	(1.47, 1.69)	<0.05
Spring	13,557	1.72	(1.60, 1.85)	<0.05
Autumn	13,647	1.78	(1.66, 1.92)	<0.05

Table S5. Univariable analysis of factors associated with timely first-dose vaccination

Variables	Univariable logistic regression analysis			
	N	Odds Ratio	Wald 95% CI	*P*-value
**Treatment regimen**				
5-dose	42,468	Reference		
4-dose	8,587	0.80	(0.75, 0.84)	<0.05
**Age**				<0.05
≤18	13,720	Reference		
(18,40]	15,755	1.07	(1.01, 1.13)	<0.05
(40,60]	13,586	1.16	(1.09, 1.23)	<0.05
＞60	7,978	1.16	(1.08, 1.25)	<0.05
**Sex**				<0.05
Female	27,043	Reference		
Male	23,996	0.94	(0.90, 0.98)	<0.05
**Ethnicity**				<0.05
Other	26,218	Reference		
Tujia	497	0.78	(0.63, 0.97)	<0.05
Han	24,238	0.94	(0.90, 0.98)	<0.05
Miao	102	1.87	(1.00, 3.49)	0.051
**Exposure site**				<0.05
Upper limbs and trunk	31,740	Reference		
Lower limbs	16,897	1.01	(0.96, 1.06)	0.77
Neck and above	1,733	0.85	(0.76, 0.96)	<0.05
**Exposure grade**				<0.05
Grade I	815	Reference		
Grade II	19,693	1.93	(1.66, 2.23)	<0.05
Grade III	30,476	3.01	(2.60, 3.49)	<0.05
**Occupational category**				<0.05
Other	17,748	Reference		
Medium-security / Market-oriented	7,401	0.94	(0.88, 1.01)	0.08
Low-security / Flexible	5,903	0.79	(0.73, 0.85)	<0.05
Economically inactive	17,246	0.86	(0.81, 0.91)	<0.05
High-security / Structured	2,741	0.91	(0.82, 1.01)	0.07
**Social identity**				<0.05
Other	29,981	Reference		
Farmer	2,917	1.18	(1.07, 1.30)	<0.05
Student	5,788	1.20	(1.11, 1.29)	<0.05
Resident	12,353	1.35	(1.27, 1.42)	<0.05
**Offending animal**				
Other or unknown animal	2,402	Reference		
Dog	25,850	0.94	(0.84, 1.04)	0.24
Cat	22,652	0.99	(0.89, 1.11)	0.88
**Use of antiserum or immunoglobulin**				<0.05
Not used	22,339	Reference		
Used	15,252	1.75	(1.66, 1.85)	<0.05
Refused	11,711	1.48	(1.40, 1.57)	<0.05
**Exposure season**				<0.05
Winter	6,720	Reference		
Summer	17,049	1.26	(1.18, 1.35)	<0.05
Spring	13,558	1.41	(1.32, 1.52)	<0.05
Autumn	13,674	1.32	(1.23, 1.42)	<0.05

Table S6. Variance inflation factors (VIF) for predictors in the multivariable logistic regression models.

Variable	GVIF	Df	GVIF(1/(2×Df))
Vaccination regimen	1.221	1	1.105
Age	2.686	3	1.179
Sex	1.029	1	1.014
Ethnicity	1.657	2	1.088
Exposure site	1.215	2	1.050
Exposure grade	2.162	2	1.212
Occupational category	1.910	4	1.084
Social identity	2.842	3	1.190
Offending animal	1.215	2	1.050
Use of antiserum/immunoglobulin	2.241	2	1.223
Season	2.034	3	1.126

Note: All adjusted GVIF^(1/(2×Df)) values ranged from 1.01 to 1.22 and were well below the recommended threshold of 2, indicating no significant multicollinearity.
